# Perceived patient burden and acceptability of MRI in comparison to PSA and ultrasound: results from the IP1-PROSTAGRAM study

**DOI:** 10.1038/s41391-023-00662-6

**Published:** 2023-03-31

**Authors:** David Eldred-Evans, Mathias Winkler, Natalia Klimowska-Nassar, Paula Burak, Martin J. Connor, Francesca Fiorentino, Emily Day, Derek Price, Martin Gammon, Henry Tam, Heminder Sokhi, Anwar R. Padhani, Hashim U. Ahmed

**Affiliations:** 1 Imperial Prostate, Division of Surgery, Department of Surgery and Cancer, Faculty of Medicine, Imperial College London, London, UK; 2 Department of Urology, Imperial College Healthcare NHS Trust, London, UK; 3 Imperial Clinical Trials Unit, Imperial College London, London, UK; 4 Division of Surgery, Department of Surgery and Cancer, Faculty of Medicine, Imperial College London, London, UK; 5 Public and patient representative, Solihull, UK; 6 Public and patient representative, Dorking, Surrey UK; 7 Department of Radiology, Imperial College Healthcare NHS Trust, London, UK; 8 Department of Radiology, The Hillingdon Hospitals NHS Foundation Trust, London, UK; 9 Paul Strickland Scanner Centre, Mount Vernon Hospital, Middlesex, UK

**Keywords:** Outcomes research, Cancer screening, Diagnostic markers, Prostate cancer

## Abstract

**Background:**

The IP1-PROSTAGRAM study showed that a short, non-contrast MRI detected more significant cancers with similar rates of biopsy compared to PSA. Herein, we compare the expected and perceived burden of PSA, MRI and ultrasound as screening tests.

**Methods:**

IP1-PROSTAGRAM was a prospective, population-based, paired screening study of 408 men conducted at seven UK primary care practices and two imaging centres. The screening tests were serum PSA, non-contrast MRI and ultrasound. If any test was screen-positive, a prostate biopsy was performed. Participants completed an Expected Burden Questionnaire (EBQ) and Perceived Burden Questionnaire (PBQ) before and after each screening test.

**Results:**

The overall level of burden for MRI and PSA was minimal. Few men reported high levels of anxiety, burden, embarrassment or pain following either MRI or PSA. Participants indicated an overall preference for MRI after completing all screening tests. Of 408 participants, 194 (47.5%) had no preference, 106 (26.0%) preferred MRI and 79 (19.4%) preferred PSA. This indicates that prior to screening, participants preferred MRI compared to PSA (+6.6%, 95% CI 4.4–8.4, *p* = 0.02) and after completing screening, the preference for MRI was higher (+21.1%, 95% CI 14.9–27.1, *p* < 0.001). The proportion of participants who strongly agreed with repeating the test was 50.5% for ultrasound, 65% for MRI and 68% for PSA. A larger proportion of participants found ultrasound anxiety-inducing, burdensome, embarrassing and painful compared to both MRI and PSA.

**Conclusions:**

Prostagram MRI and PSA are both acceptable as screening tests among men aged 50–69 years. Both tests were associated with minimal amounts of anxiety, burden, embarrassment and pain. The majority of participants preferred MRI over PSA and ultrasound.

**Registration:**

This study was registered on clinicaltrials.gov at https://clinicaltrials.gov/ct2/show/NCT03702439.

## Introduction

The acceptability of a screening test is an important determinant of participation and, ultimately, the effectiveness of a screening programme [1]. The impact of attendance rates has been demonstrated in micro-simulation models within the European prostate screening study in which the relative reduction in prostate cancer mortality was almost eliminated when attendance to PSA screening was reduced to 30% [2]. Indeed, in colorectal cancer screening, even though faecal occult blood testing was effective at reducing mortality [3], the uptake rates were sub-optimal when rolled out as a national screening test. The introduction of the faecal immunochemical test (FIT), which was easier to complete and perceived as less unpleasant, led to an increase in participation [4].

We recently published results of the IP1-PROSTAGRAM study in which the role of a short MRI or transrectal ultrasound, akin to mammography for breast cancer, was used to determine if they might be better screening tests than PSA. Our primary results showed that a Prostagram MRI found more clinically significant cancers at a similar rate of biopsies compared to PSA. Ultrasound had a high degree of false positives [5]. There have been no previous studies comparing the acceptability of men undergoing these tests for prostate cancer. Studies in colorectal cancer screening has shown that acceptability can be related to the expected and perceived burden of screening using domains such as the expected level of embarrassment or pain [6, 7]. We compared the expected and perceived burden of PSA, MRI and ultrasound as screening tests.

## Methods

This study was embedded in the IP1-PROSTAGRAM trial [5]. In summary, men based in the community who expressed an interest in participating were informed of a series of tests to screen for prostate cancer. The initial invitation referred to blood tests and imaging tests. It did not incorporate extensive details regarding each screening test to minimise selection bias, reflecting the phased approach to information giving used in other studies, such as the UK lung health check programme [8].

### Participants

For the present study, we selected participants who completed acceptability questionnaires before and after each test. Screening tests were performed in a fixed order; PSA, ultrasound and then MRI. All responders received a detailed information leaflet to provide more information about each screening procedure and to facilitate informed decision-making, which were written and reviewed by GPs, urologists, radiologists, expert patient advisers and the Patient and Public Involvement panel. Research Ethics Committee approval was given (ref: 8/LO/1338).

### Questionnaires

Participants completed the Expected Burden Questionnaire (EBQ) and Perceived Burden Questionnaire (PBQ) adapted for this study [9].

#### Expected burden questionnaire (EBQ)

Participants completed the EBQ questionnaire in the waiting area prior to having any screening test. The EBQ is comprised of four domains addressing the expected extent of embarrassment, pain, burden and anxiety caused by each test. This is followed up by a question summarising which test the patient expects to prefer. All items are on a 5-point LIKERT scale (1 = not at all, 2 = slightly, 3 = somewhat, 4 = rather, 5 = extremely).

#### Perceived burden questionnaire (PBQ)

After each screening test, participants completed a second questionnaire in the waiting area immediately after each test without knowing the results. Similar to the EBQ, the PBQ is comprised of five questions addressing the embarrassment, pain, burden and anxiety experienced from each test and how likely the patient was to have the test again, if recommended. This was followed up by a question summarising which test the patient preferred. For each of the screening tests, 5-point Likert scales were used to elicit the subject’s perception of anxiety, burden, embarrassment and pain.

### Statistical analysis

Statistical analysis was performed using R version 2.3.1 (R Foundation for Statistical Computing). None of the variables were found to satisfy assumptions of normality required for paired t-tests despite multiple transformations, including log transformation. Due to the paired nature of the data, the primary analysis used a two-sample paired sign test which is not limited by the distributional assumptions of the parametric paired samples t-test. The Sign test looks specifically at the median value of differences and is not affected by the distribution of the data. The null hypothesis of the two-sample paired Sign test is that the median of the difference is zero. Each question, representing each domain, is reported separately. An overall burden score was calculated for each screening test by summing the response scores to the first four questions, excluding the question relating to repeat test recommendation. Lower overall scores represent a lower perceived overall burden for that particular screening test.

Secondary analyses used the Wilcoxon ranked sign tests. The Likert scores were assumed to be a continuous distribution to allow a comparison of the mean scores for anxiety, burden, pain and embarrassment. Sankey charts were adopted to visually show the transitions of EBQ and PBQ scores between screening tests. This paired analysis included only men who completed both questionnaires. Spearman’s rank correlation rho was performed to determine the correlation between pre and post-scores. Differences in overall preference between tests were compared with the Chi-squared test.

A series of univariable regression analyses were conducted to examine the association between predictors and overall PBQ score. The overall PBQ was dichotomised by the median value for each screening test, so that the outcome variable was binary. Covariates were divided into three groups which include baseline factors such as age (continuous), Afro-Caribbean ethnicity, Index of Multiple Deprivation (IDDM), Qualification at Degree, A-level or equivalent, Employment Status, Family history of prostate cancer and previous screening for prostate cancer. The IDDM is an area-based proxy for socio-economic status and was dichotomised into the least/most deprived compared to others. Psychological factors included pre-test levels of expected anxiety, burden, embarrassment and pain as measured by the EBQ. Procedure factors included variables which might affect the degree of burden for each test and included length of procedure, body mass index and prostate volume for MRI only. Multivariable binary logistic regression was then performed for all variables in the univariable analysis with a significant or near significance *p* < 0.1. Statistically, significance was set a *p* < 0.05.

## Results

### Study population

Between October 2018 and May 2019, a total of 411 men aged 50–69 years attended for screening. Prior to the screening, 403/408 (98.7%) completed each domain of the pre-test EBQ. All men completed the PSA screening test and the post-test questionnaire (PBQ) and one participant did not complete the post-test MRI questionnaire (407/408, 99.7%). The mean duration in minutes for each screening test was 1.3 for PSA (SD 0.59), 19.7 for MRI (SD 3.83) and 8.4 for ultrasound (SD 3.17).

### Outcomes

**Fig. 1:**
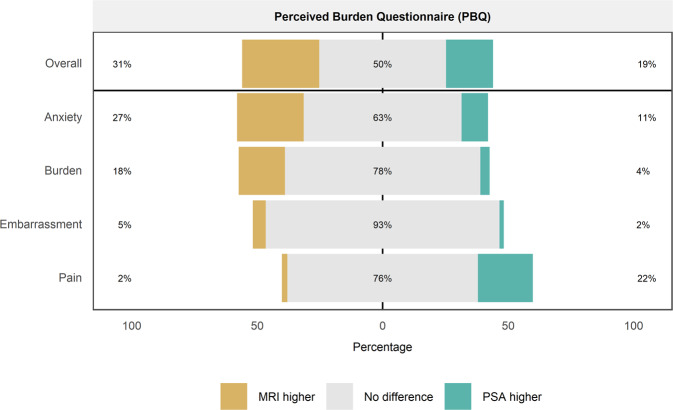
Comparison of PBQ Scores for MRI vs PSA. Diverging bar chart depicting the difference between participantʼs anxiety, burden, embarrassment and pain for PSA and MRI.

The perceived overall burden for MRI and PSA were compared. In total, 30.8% perceived MRI to be worse than PSA, 18.7% perceived MRI to be better than PSA, and 50.4% perceived MRI and PSA to be the same (Table 1); PSA was perceived to have a lower burden than MRI (*p* = 0.0007). In terms of the component scores, 108 (26.5%) had higher anxiety scores for MRI than PSA, 43 (10.6%) had a score for MRI lower than PSA, and 256 (62.9%) had equal scores (*p* < 0.001). For the burden component, 75 (18.5%) perceived MRI to be more burdensome than PSA, 15 (3.7%) perceived PSA to be more burdensome than MRI, and 316 (77.8%) perceived them to be similar (*p* = 0.001). The embarrassment component showed 21 (5.2%) perceived MRI as more embarrassing than PSA, 7 (1.7%) perceived PSA as more embarrassing than MRI, and 379 (93.1%) perceived both as similar embarrassment. The pain component was the only score which was higher for PSA, with 9 (2.2%) perceiving MRI to be more painful, 89 (21.9%) PSA to be more painful, and 75.9% to be similar (*p* < 0.0001) (Fig. 1).

**Table 1:** Output of non-parametric two-sample paired Sign test comparing PBQ scores for MRI vs. PSA.

	MRI + ve	PSA + ve	No difference	*P* value (*n*)
Anxiety	108 (26.5%)	43 (10.6%)	256 (62.9%)	<0.0001*** (*n* = 151)
Burden	75 (18.5%)	15 (3.7%)	316 (77.8%)	<0.0001*** (*n* = 90)
Embarrassment	21 (5.2%)	7 (1.7%)	379 (93.1%)	0.013* (*n* = 28)
Pain	9 (2.2%)	89 (21.9%)	309 (75.9%)	<0.0001*** (*n* = 98)
Overall	125 (30.8%)	76 (18.7%)	205 (50.5%)	0.0007*** (*n* = 201)

^*^Significant at 0.05 level (two-sided), ***Significant at 0.001 level (two-sided).

#### Mean scores and Wilcoxon comparison

##### EBQ

(Table 2) The overall mean EBQ score for MRI and PSA was 1.45 (SD 0.65) and 1.46 (SD 0.54), respectively. Comparison between individual domain scores showed that a higher proportion having MRI expected it to have more anxiety (1.75 vs 1.63, *p* = 0.02), more burdensome (1.47 vs 1.33, *p* < 0.001) and more embarrassing. The expected pain score for PSA was higher than MRI (1.67 vs. 1.26, *p* < 0.001).

**Table 2:** Output of Wilcoxon signed-rank test to compare mean EBQ & PBQ scores.

	MRI	PSA	*P* value
Expected Burden (EBQ)			
Anxiety	1.75 (1.00)	1.63 (0.86)	0.02
Burden	1.47 (0.76)	1.33 (0.65)	<0.001
Embarrassment	1.32 (0.65)	1.21 (0.55)	<0.001
Pain	1.26 (0.67)	1.67 (0.72)	<0.001
Overall	1.45 (0.65)	1.46 (0.54)	0.027
Perceived Burden (PBQ)			
Anxiety	1.46 (0.77)	1.26 (0.65)	<0.001
Burden	1.29 (0.62)	1.11 (0.45)	<0.001
Embarrassment	1.08 (0.38)	1.03 (0.26)	0.03
Pain	1.04 (0.20)	1.25 (0.48)	<0.001
Overall	1.21 (0.35)	1.16 (0.36)	0.003

Figures are *n* (SD).

##### PBQ

The participants perceived the overall burden of MRI to be higher than PSA (1.21 vs. 1.16, *p* = 0.003) (Table 2). A comparison of the component scores showed that the difference in overall score was due to a higher degree of anxiety (1.46 vs. 1.26, *p* < 0.001), burden (1.29 vs. 1.11, *p* < 0.001) and embarrassment (1.08 vs. 1.03, *p* = 0.03). The pain score remained higher for PSA compared to MRI (1.25 vs. 1.04, *p* < 0.001). All component scores were lower in the PBQ compared to the EBQ.

#### MRI comparison of expected and perceived scores

The participants’ transition between EBQ (pre-test) and PBQ (post-test) for MRI are shown in Sankey Diagrams (Fig. S1). There was a consistent trend for men to have lower pre-test scores across all component scores. For anxiety, 16% expected some/rather/extreme anxiety-reducing to 7% (Spearman’s rank correlation 0.421, *p* < 0.001); no burden increased from 65 to 78% (Spearman’s rank correlation 0.227, *p* < 0.001); for embarrassment, 6% expected it to have some/rather/extreme embarrassment but reducing to 2%; and for pain 5 to 1% (Spearman’s rank correlation coefficients of 0.141 and 0.103 for embarrassment and pain, *p* < 0.001).

#### PSA comparison of expected and perceived scores

There was a similar trend for PSA, with post-test (PBQ) scores improving compared to pre-test scores (Fig. S2). For anxiety, the proportion of participants who reported no anxiety increased from 54 to 82% (Spearman’s rank correlation 0.376, *p* < 0.001); no burden increased from 74 to 92%; no embarrassment was 98% after the PSA test (Spearman’s rank correlation coefficients of 0.325 and 0.334 for burden and pain, *p* < 0.001); 56% who expected the phlebotomy procedure to be associated with slight/some/rather/extreme pain reduced to 23% (Spearman’s rank correlation 0.334, *p* < 0.001).

#### Preference for screening examination

**Fig. 2:**
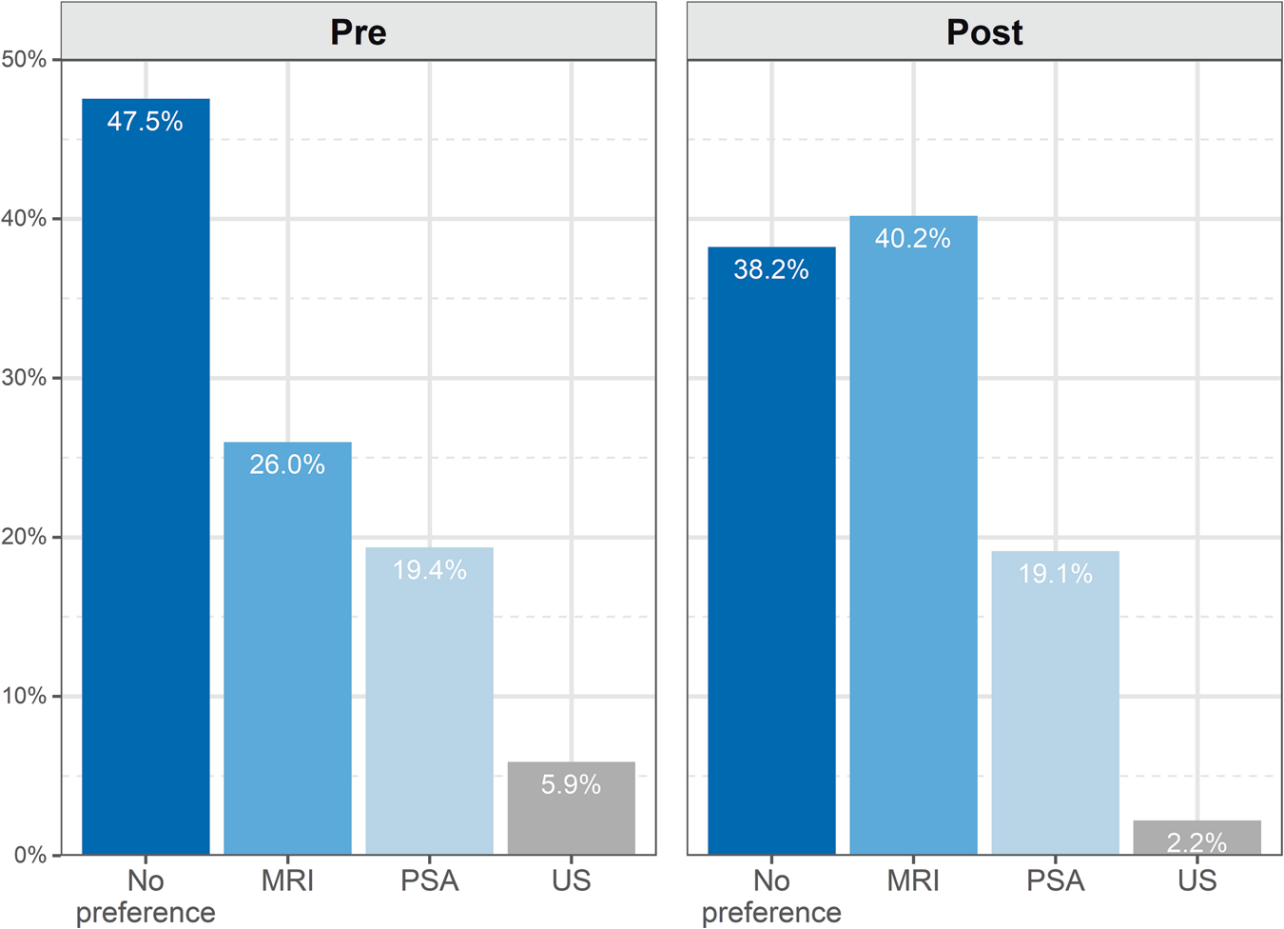
Overall preference of participants for the screening test. Bar chart illustrating that participants indicated an overall preference for MRI after completing all screening tests.

Of 408 participants, 194 (47.5%) had no preference, 106 (26.0%) preferred MRI and 79 (19.4%) preferred PSA. This indicates that prior to screening, participants preferred MRI compared to PSA (+6.6%, 95% CI 4.4–8.4, *p* = 0.02). The proportion of participants who had no preference was higher than any category (*p* < 0.001) (Fig. 2). After undergoing all screening tests, 164 (40.2%) preferred MRI, 156 (38.2%) had no preference and 78 (19.1%) preferred PSA. The proportion of participants who preferred MRI compared to PSA was +21.1% (95% CI 14.9–27.1, *p* < 0.001).

### Determinants of the overall burden

#### MRI

In the multivariable regression analysis, the presence of pre-test anxiety (odds ratio 2.16, *p* < 0.001) and Afro-Caribbean ethnicity (odds ratio 0.521, *p* = 0.048) were identified as significant determinants of the overall burden of MRI (Table S3).

#### PSA

Multivariable analysis demonstrated that the presence of pre-test anxiety (OR 1.12, *p* < 0.001) and pre-test expected burden (OR 1.14, *p* < 0.001) were identified as significant determinants of overall burden (Table S4).

### Acceptability of ultrasound

#### Expected and perceived scores

For ultrasound, the majority of post-test domains were lower than the pre-test scores indicating participants found the ultrasound test more burdensome than expected (Fig. S3).

#### Comparison with other tests

**Fig. 3:**
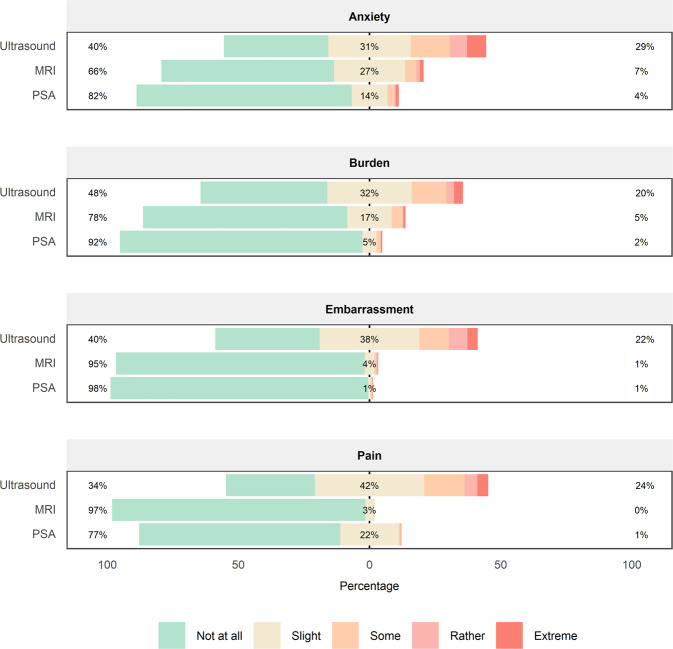
Distribution of PBQ Scores for Ultrasound, MRI and PSA. Bar chart depicting participantʼs perception of anxiety, burden, embarrassment and pain after having a Ultrasound, MRI and PSA.

A comparison of the post-test (PBQ) scores of the three screening tests showed a larger proportion of participants found ultrasound anxiety-inducing, burdensome, embarrassing and painful compared to both MRI and PSA (Fig. 3).

#### Determinants of ultrasound burden

Multivariable regression analysis demonstrated Afro-Caribbean ethnicity (OR 2.10, *p* = 0.043) and pre-test anxiety (OR 2.16, *p* < 0.001) as significant determinants of the overall burden of ultrasound (Table S5).

#### Willingness to repeat ultrasound

In total, 34.6% (95% CI 17.1–24.9) either strongly disagreed, disagreed or were neutral with a recommendation for a repeat ultrasound in comparison to 22.9% (95%CI 19.1–27.2) and 24.6% (95% CI 20.7–29.0) for PSA and MRI, respectively (Fig. S7).

## Discussion

Our analysis focused on patient-reported experience measures related to each screening test, with more than 6483 responses across the EBQ and PBQ domains. We showed that the overall level of burden for MRI and PSA was minimal. Few men reported high levels of anxiety, burden, embarrassment or pain following either test. Participants indicated an overall preference for MRI after completing all screening tests. Ultrasound was perceived and experienced as having the worse overall burden.

Key strengths of this study are the high response rate, well-balanced cohort, paired design and use of validated patient-reported outcomes measures. The paired design allowed a direct comparison between the tests and the response rate to both questionnaires was above 98%. The generalisability of the results was enhanced by the broad representation of ethnic groups, socio-economic status and educational backgrounds within IP1-PROSTAGRAM in contrast to previous studies, which have recruited predominantly Caucasian males (Table S1).

Although the differences between MRI and PSA were statistically significant, the actual differences were often small when considered relative to the score within each domain. Previous studies have suggested that the threshold for clinically important differences in such questionnaires is ~0.5 SD [10]. In this analysis, the pooled SD was 0.35 for MRI and 0.36 for PSA for overall scores, suggesting that the variation in scores is unlikely to have a clinically significant impact on the majority of participants.

Participants reported that the transrectal ultrasound had a higher level of burden, embarrassment and pain than expected and were less willing to accept a repeat examination compared to MRI. The comparison of post-test scores showed that ultrasound had lower scores across all domains. Other screening tests, such as flexible sigmoidoscopy with a rectal approach, similarly lead to low acceptability and reduced attendance [11–13].

An interesting finding was that although the levels of embarrassment and pain were high for ultrasound, half of the participants would strongly agree to repeat the examination. This was not the case for the majority of Afro-Carribean participants, who found the ultrasound more burdensome. The finding that Afro-Caribbean ethnicity is associated with a higher burden is significant given the importance of finding an appropriate screening test in this demographic due to the higher risk of prostate cancer. This supports previous studies which have shown this as a particular barrier for this group due to a culturally linked aversion to rectal examination [14, 15]. This is an important finding as we would want a screening programme to avoid amplifying existing disparities in access to healthcare, particularly as this demographic are at higher risk of prostate cancer.

There is a paucity of published data comparing patient-reported outcomes for PSA and MRI as screening tests. There have been a few studies focused on anxiety levels in men with an abnormal PSA test which have generally found that receipt of an abnormal test did not have a significant effect on anxiety levels [16, 17]. Our findings are comparable, if not with lower burden scores, to other screening studies into CT-colonoscopy for bowel cancer screening [18].

The multivariate analysis found that expected anxiety and/or burden are important patient-related determinants of screening test experience. The fact that pre-test perceptions of the test strongly influenced the experience indicates that more intensive efforts to address preconceptions and patient-related anxiety for each test are needed. The participants in IP1-PROSTAGRAM received extensive explanation and discussions as part of the consent process, and it is less likely that improvements can be made by providing more verbal or written or visual (e.g. video) information prior to MRI. Other screening tests, such as low-dose CT for lung cancer, have implemented alternative strategies [19]. Video or online interventions can be effective at improving anxiety and knowledge prior to imaging tests [20]. This is important given that patient experience of a screening test is an important determinant of future and ongoing participation with multiple rounds of screening [21].

There are a number of areas which could be considered to reduce the overall burden of MRI. The MRI protocol in IP1-PROSTAGRAM was set up to be around 15 min, but since the development of the protocol, there are several faster techniques which have been developed to reduce scanning time without impacting diagnostic accuracy [22–24]. In addition, a more acceptable gown than the use of a hospital-provided gown for the MRI might minimise embarrassment.

There are some limitations. First, EBQ and PBQ questionnaires have been validated for colorectal cancer screening, so were adapted by this study for prostate cancer screening tests, given the lack of validated questionnaires designed for prostate cancer screening tests. Second, when determining overall preference for screening tests, participants were given no information on the costs or predicted accuracy of the tests; diagnostic accuracy and cost are important factors in patient preference of screening test choice [25]. Third, the overall preference responses were recorded immediately before and after each screening test, as has been done in other studies. However, other studies have shown that preferences may change over time [26] with less pronounced preferences, although participants might find it difficult to differentiate tests afterwards [27]. Fourth, recruitment included volunteers and men with claustrophobia were excluded from the protocol. We attempted to minimise selection bias of the cohort through the design of recruitment materials. The outcomes of our recruitment have been published elsewhere and show that participants who accepted invitations were older than non-responders (58.9 years vs 57.2 years) [28]. It is accepted that this difference may influence the tolerability of the screening tests in our cohort. Fifth, the non-random order of the tests may have introduced bias in the participants’ answers. The protocol stipulated a fixed order to ensure that the PSA test was performed before the ultrasound examination which can induce false PSA elevation.

## Conclusion

Prostagram MRI and PSA are both acceptable as screening tests for men aged 50–69 years. Both tests were associated with minimal amounts of anxiety, burden, embarrassment and pain. The majority of participants preferred MRI over PSA and ultrasound.

## Supplementary information

Supplementary Material

## Data Availability

The datasets used and/or analysed during the current study are available from the corresponding author on reasonable request.
