# Supplementary material for: Perceived patient burden and acceptability of MRI in comparison to PSA and ultrasound: results from the IP1-PROSTAGRAM study
Source: Prostate Cancer Prostatic Dis. 2023 Mar 31;26(3):531–7. doi: 10.1038/s41391-023-00662-6 (PMC10449626; doi:10.1038/s41391-023-00662-6)
Supplement: Supplementary file 1 — Supplementary Material [file 41391_2023_662_MOESM1_ESM.docx]

**Supplementary Figure 1: Sankey chart for MRI showing the relationship between the pre-test (EBQ) and post-test scores. Within each panel the left bar chart represents the pre (EBQ) score and the right represents the post-screening score. The ribbons connecting the left and right axis are proportional to the number of participants who transition from the pre-score to the post-score. Labels are shown for proportions above 2%.**

***
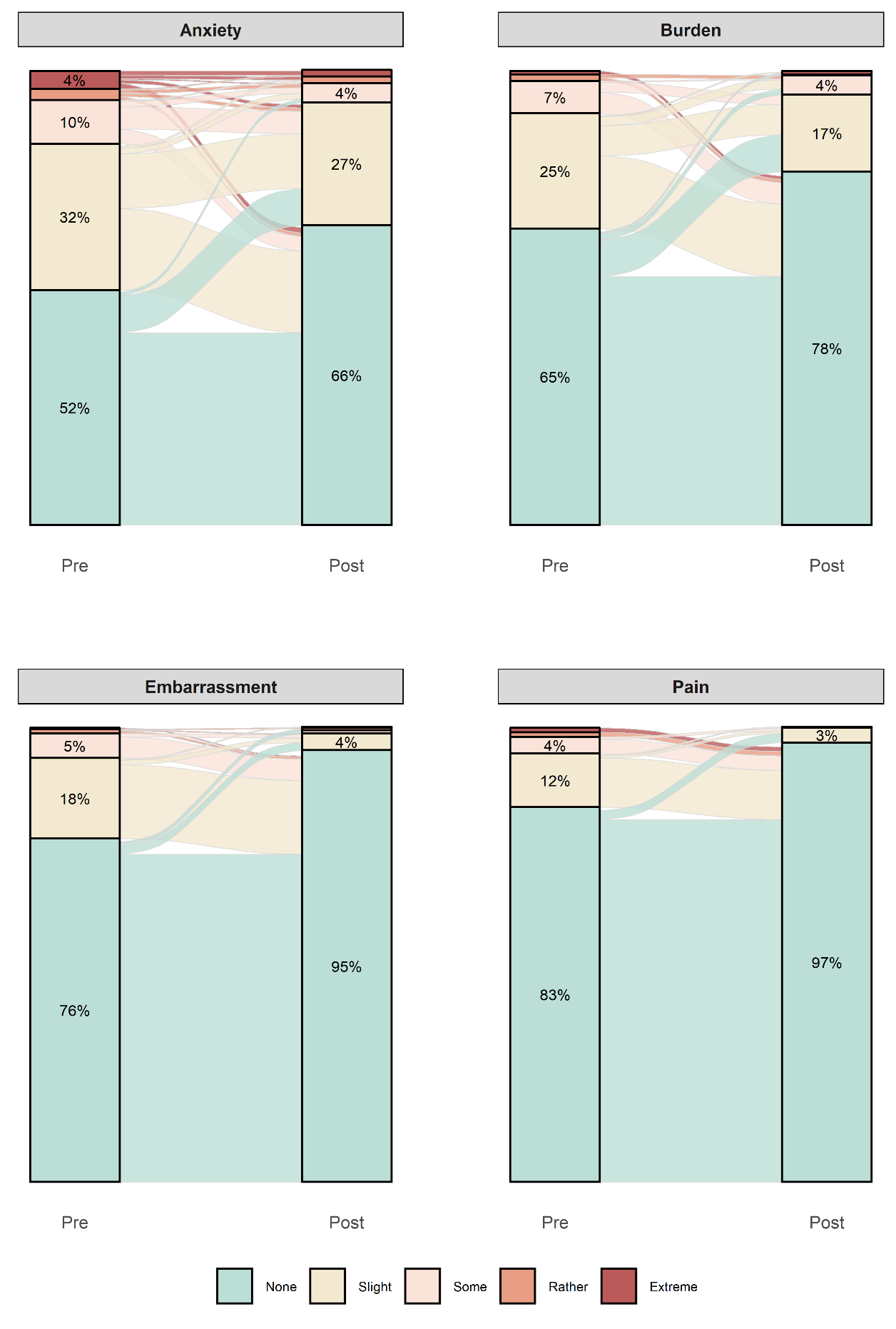
***

**Supplementary Figure 2: Sankey chart for PSA showing the relationship between the pre-test scores and post-test scores. Labels are shown for proportions above 2%.**

***
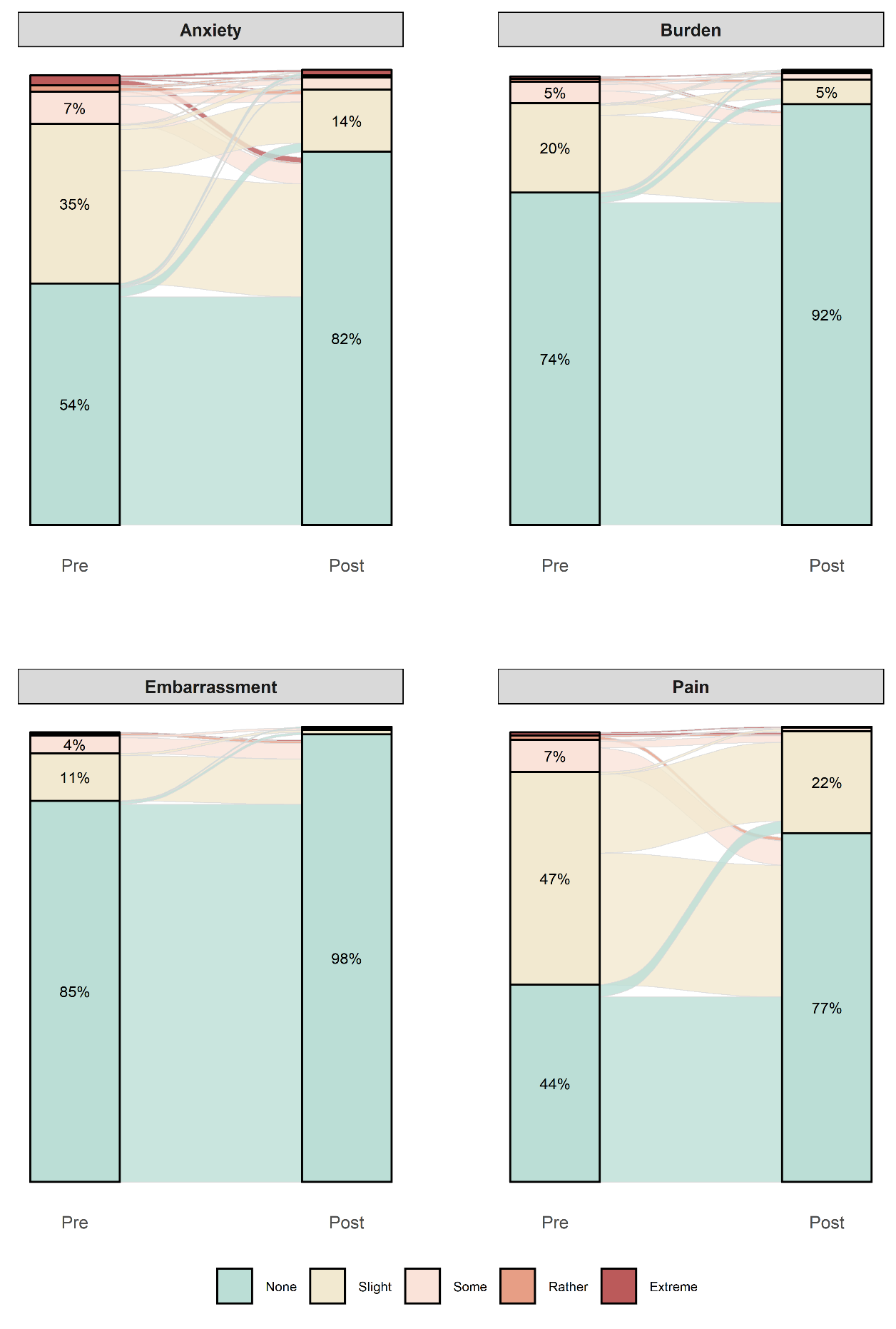
***

**Supplementary Figure 3: Sankey chart for US showing the relationship between the pre-test scores and post-test scores. Labels are shown for proportions above 2%.**

***
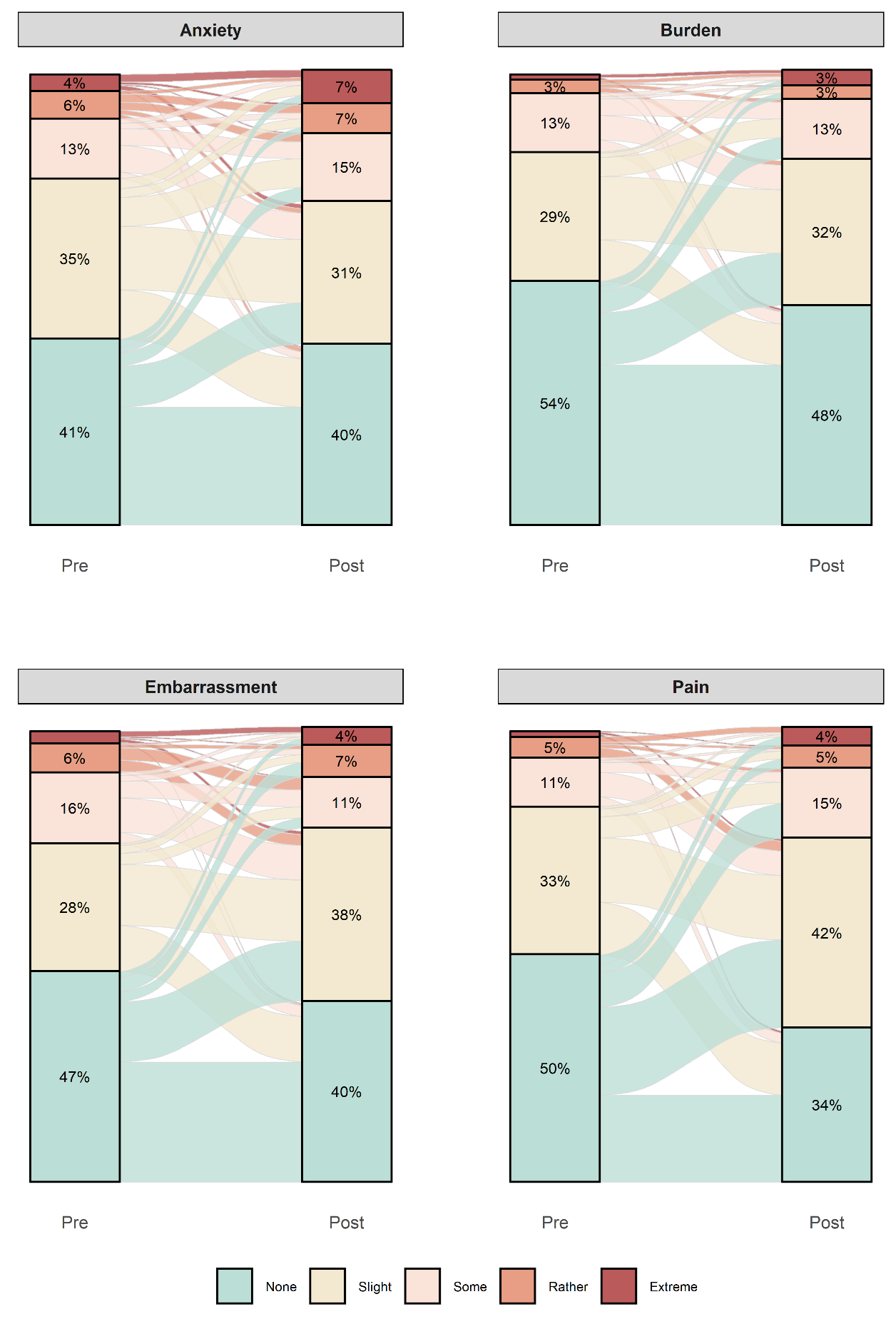
***

**Supplementary Figure 4: Divergent stacked bar charge showing the PBQ components for MRI vs US**

**
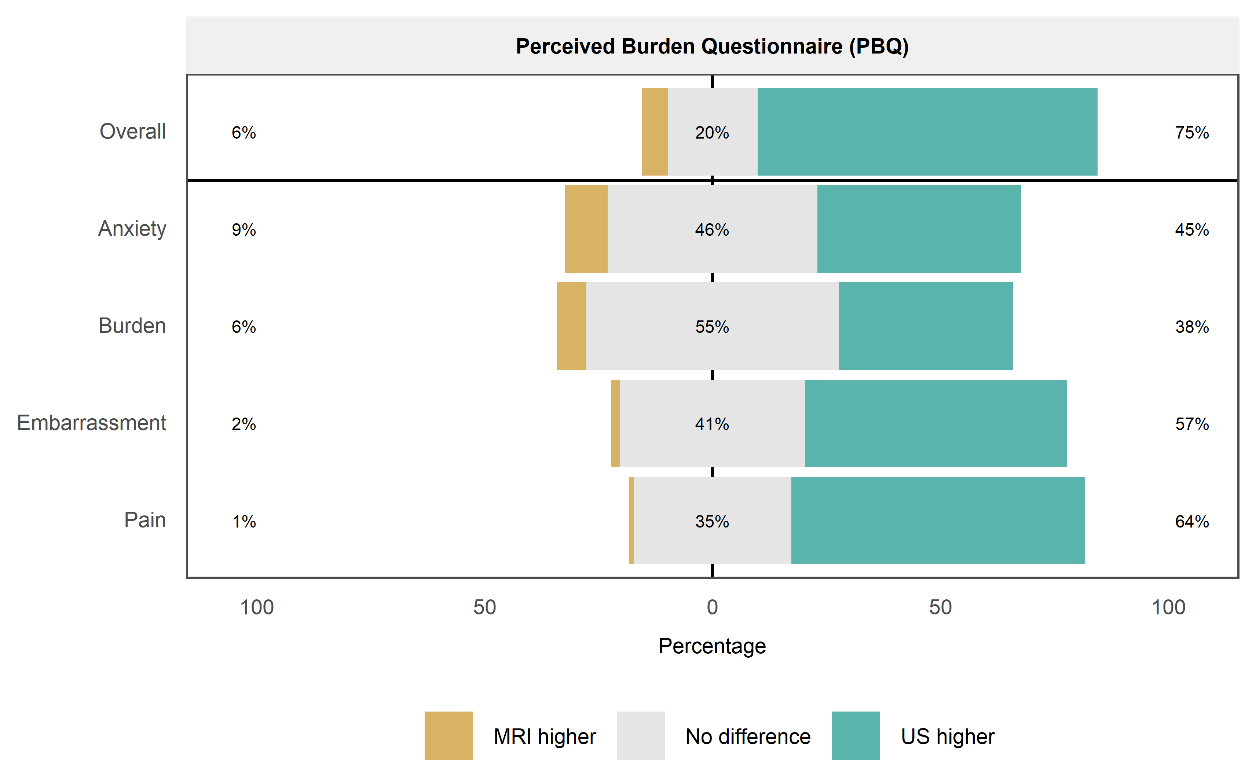
**

**Supplementary Figure 5: Divergent stacked bar charge showing the PBQ components for PSA vs US**

***
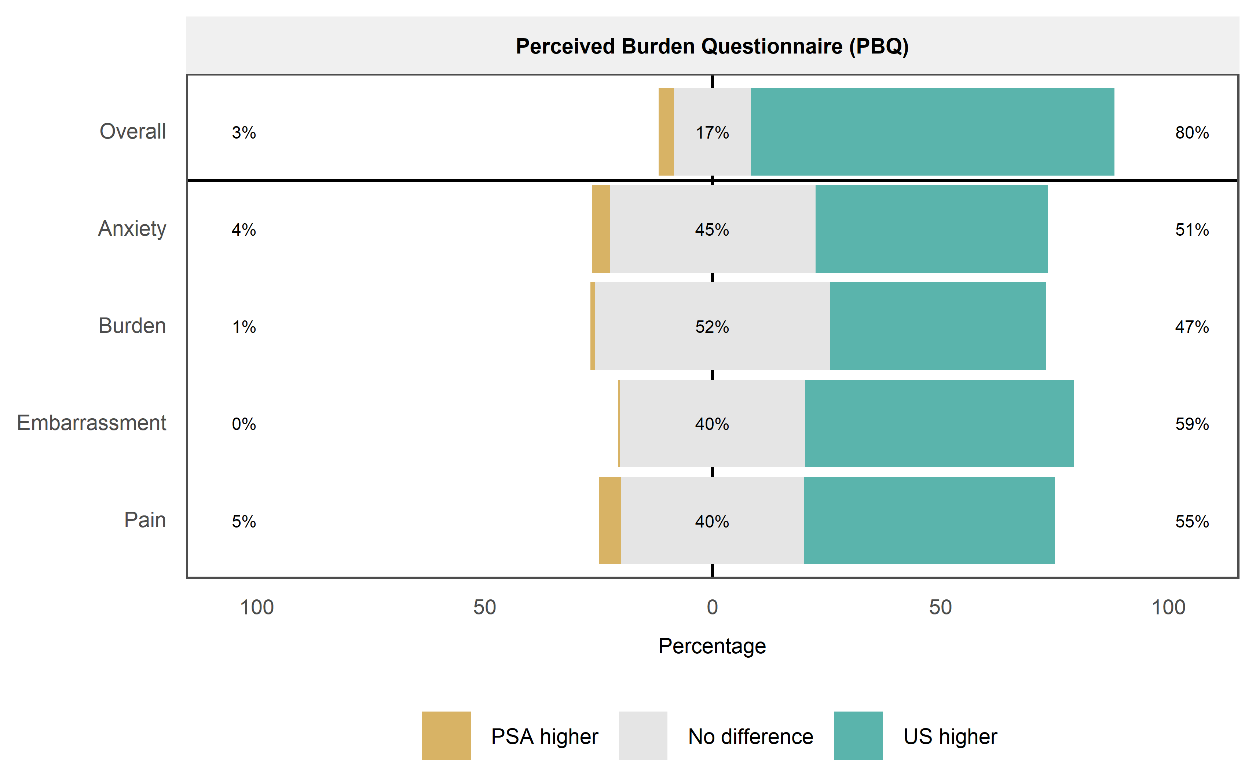
***

**Supplementary Figure 6: Sankey flow diagram focusing on the flow of overall preference before and after completing each screening test.**


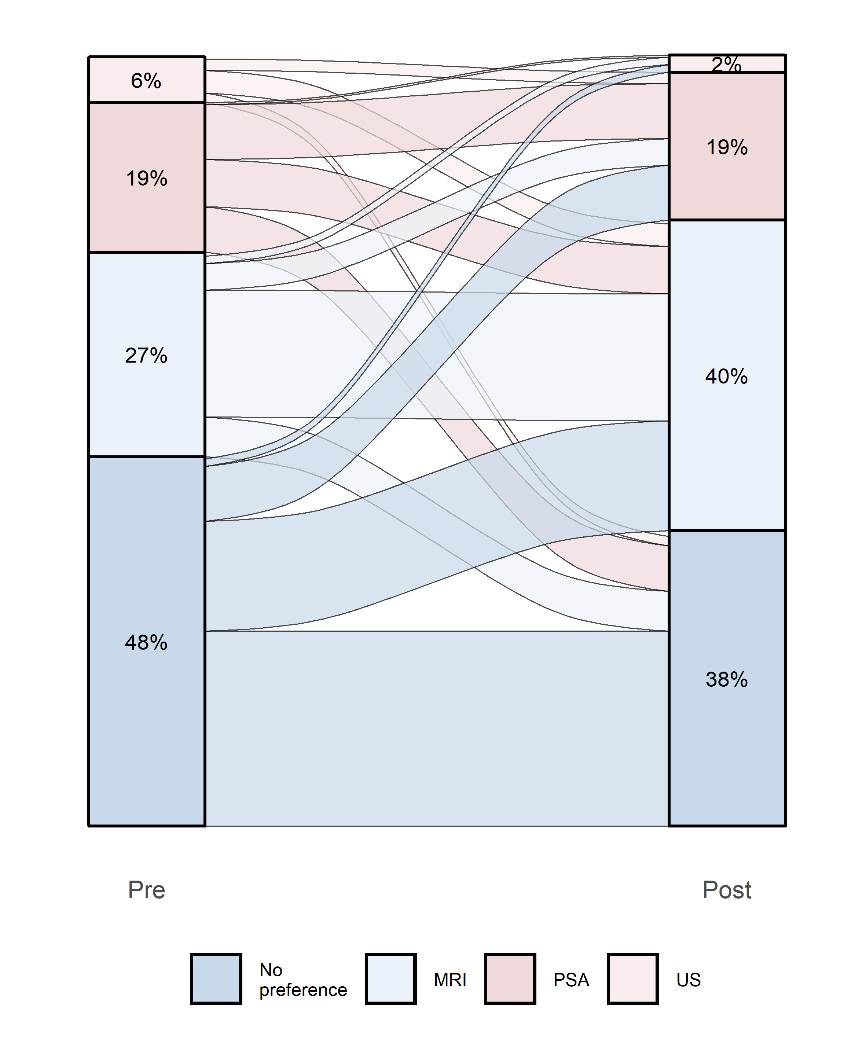


**Supplementary Figure 7: A bar chart comparing the willingness of participants to have a repeat ultrasound, MRI or PSA screening test.**


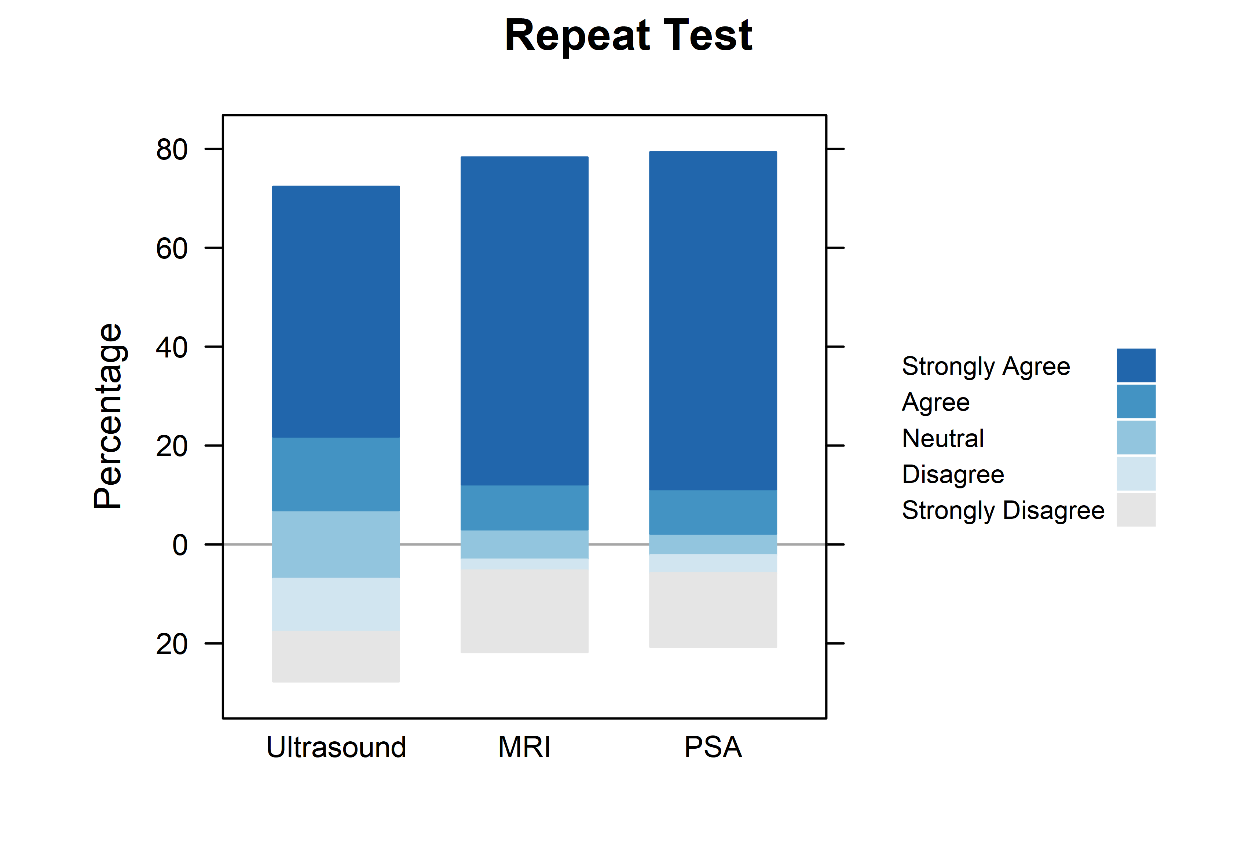


**Supplementary Tabe 1: Demographics**

|  | **Participants (n = 408)** |
| --- | --- |
| **Age (years)** | |
| 50-54 | 140 (34.3%) |
| 55-59 | 127 (31.1%) |
| 60-64 | 85 (20.8%) |
| 65-69 | 56 (13.7%) |
| **Ethnicity** | |
| White | 155 (38.0%) |
| Black | 132 (32.4%) |
| Asian | 94 (23.0%) |
| Other | 18 (4.4%) |
| Mixed race | 9 (2.2%) |
| **Index of Multiple Deprivation Quintile** | |
| 1 (least deprived) | 54 (13.2%) |
| 2 | 109 (26.7%) |
| 3 | 137 (33.6%) |
| 4 | 62 (15.2%) |
| 5 (most deprived) | 44 (10.8%) |
| Unknown | 2 (0.5%) |
| **Charlson Comorbidity** | |
| 0 (none) | 324 (79.4%) |
| 1 (mild) | 49 (12.0%) |
| ≥ 2 (severe) | 13 (3.2%) |
| Unknown | 22 (5.4%) |
| **First degree relative with prostate cancer** | |
| Yes | 43 (10.5%) |
| No | 360 (88.2%) |
| Unknown | 5 (1.2%) |
| Data are n (%).  PSA = Prostate-specific antigen | |

**Supplementary Table 2: Output of non-parametric two-sample paired Sign test comparing EBQ scores for MRI vs. PSA**

|  | | | | | | |
| --- | --- | --- | --- | --- | --- | --- |
|  | **MRI +ve** | **PSA +ve** | **No difference** | **P-value (n)** | |  |
| Anxiety | 87 (21.6%) | 70 (17.4%) | 246 (61.0%) | 0.202  (n=157) | |  |
| Burden | 86 (21.4%) | 51 (12.7%) | 265 (65.9%) | 0.0035**  (n=137) | |  |
| Embarrassment | 76 (18.9%) | 37 (9.2%) | 290 (72.0%) | 0.0003***  (n=113) | |  |
| Pain | 28 (6.9%) | 178 (44.2%) | 197 (48.9%) | <0.0001***  (n=206) | |  |
| Overall | 103 (25.6%) | 143 (35.6%) | 143 (35.6%) | 0.013*  (n = 246) | |  |
| *Significant at 0.05 level (two-sided), ***Significant at 0.001 level (two-sided) | | | | |  |  |

**Supplementary Table 3:** **Univariate and Multi-variate analysis for overall burden of MRI**

|  | Univariate Analysis | | Multivariate Analysis | | |  |
| --- | --- | --- | --- | --- | --- | --- |
| **Covariate** | **OR (95% CI)** | **p value** | **OR (95% CI)** | **p value** |  |  |
| **Baseline Factors** |  |  |  |  |  |  |
| Age | 1.02  (0.99 - 1.04) | 0.232 | - | - |  |  |
| Afro-Caribbean Ethnicity | 0.62  (0.35 - 1.06) | 0.086 | 0.521  (0.27-0.98) | **0.048** |  |  |
| Index of Deprivation* | 1.20  (0.80 - 1.81) | 0.412 | - | - |  |  |
| Qualification Level** | 1.02  (0.76-1.41) | > 0.9 | -  - | - |  |  |
| Employment Status¥ | 1.02  (0.74-1.41) | >0.9 | - | - |  |  |
| Family history^ | 1.23  (0.79-1.92) | 0.434 | - | - |  |  |
| Previous screening^^ | 1.19  (0.90-1.58) | 0.243 | - | - |  |  |
|  |  |  |  |  |  |  |
| **Psychological Factors** | | | | |  |  |
| Expected Anxiety | 1.75  (1.54-1.98) | <0.001 | 2.16  (1.44–3.33) | **<0.001** |  |  |
| Expected Burden | 1.74  (1.46-2.06) | <0.001 | 0.979  (0.784–2.42) | 0.26 |  |  |
| Expected Embarrassment | 1.60  (1.31-1.97) | <0.001 | 0.485  (0.485-1.53) | 0.64 |  |  |
| Expected Pain | 1.93  (1.59-2.34) | <0.001 | 0.725  (0.725-2.25) | 0.41 |  |  |
|  |  |  |  |  |  |  |
| **Procedural Factors** | | | | |  |  |
| MRI Procedure Length | 1.02  (0.98-1.06) | 0.312 | - | - |  |  |
| BMI | 1.01  (0.98-1.05) | 0.434 | - | - |  |  |
| Prostate Volume | 0.99  (0.98-1.01) | 0.332 | - | - |  |  |
| - Insufficient association on univariate analysis to continue in the multivariate analysis  * Most Deprived (Quintile 5) vs. Other, ** A-Level, Degree or Equivalent vs. lower/none  ¥ Employed vs. any other. ^ First or second degree relative with prostate cancer,  ^^ Defined as either PSA or DRE screening | | | | | | |

**Supplementary Table 4: Univariate and Multi-variate analysis for overall burden of PSA**

|  | Univariate Analysis | | Multivariate Analysis | | |  |
| --- | --- | --- | --- | --- | --- | --- |
| **Covariate** | **OR (95% CI)** | **p value** | **OR (95% CI)** | **p value** |  |  |
| **Baseline Factors** |  |  |  |  |  |  |
| Age | 1.00  (0.97-1.02) | 0.812 | - | - |  |  |
| Afro-Caribbean Ethnicity | 1.58  (1.19-2.10) | 0.002 | 1.03  (0.95-1.12) | 0.47 |  |  |
| Index of Deprivation* | 0.83  (0.54-1.29) | 0.454 | - | - |  |  |
| Qualification Level** | 1.05  (0.80-1.39) | 0.712 | 0.963  (0.86-1.01) | 0.08 |  |  |
| Employment Status¥ | 0.97  (0.71-1.33) | 0.823 | - | - |  |  |
| Family history^ | 0.77  (0.50-1.19) | 0.213 | - | - |  |  |
| Previous screening^^ | 1.20  (0.92-1.59) | 0.242 | - | - |  |  |
|  |  |  |  |  |  |  |
| **Psychological Factors** | | | | |  |  |
| Expected Anxiety | 1.84  (1.59-2.13) | <0.001 | 1.12  (1.06-1.18) | **<0.001** |  |  |
| Expected Burden | 1.94  (1.59-2.37) | <0.001 | 1.14  (1.06-1.22) | **<0.001** |  |  |
| Expected Embarrassment | 1.56  (1.23-2.00) | <0.001 | 0.959  (0.89-1.03) | 0.19 |  |  |
| Expected Pain | 1.97  (1.65-2.35) | <0.001 | 1.05  (0.98-1.12) | 0.17 |  |  |
|  |  |  |  |  |  |  |
| **Procedural Factors** | | | | |  |  |
| PSA Procedure Length | 1.11  (0.88-1.41) | 0.412 | - | - |  |  |
| BMI | 0.98  (0.95-1.02) | 0.343 | - | - |  |  |
| - Insufficient association on univariate analysis to continue in the multivariate analysis  * Most Deprived (Quintile 5) vs. Other, ** A-Level, Degree or Equivalent vs. lower/none  ¥ Employed vs. any other. ^ First or second degree relative with prostate cancer,  ^^ Defined as either PSA or DRE screening | | | | | | |

**Supplementary Table 5: Univariate and Multi-variate analysis for overall burden of Ultrasound**

|  | Univariate Analysis | | Multivariate Analysis | | |  |
| --- | --- | --- | --- | --- | --- | --- |
| **Covariate** | **OR (95% CI)** | **p value** | **OR (95% CI)** | **p value** |  |  |
| **Baseline Factors** |  |  |  |  |  |  |
| Age | 0.93  (0.87-1.00) | 0.040 | 0.99  (0.926-1.05) | 0.69 |  |  |
| Afro-Caribbean Ethnicity | 2.33  (1.11-4.92) | 0.027 | 2.10  (1.02-4.30) | **0.043** |  |  |
| Index of Deprivation* | 0.99  (0.32-3.08) | 0.98 | - | - |  |  |
| Qualification Level** | 0.69  (0.33-1.46) | 0.33 | -  - | - |  |  |
| Employment Status¥ | 1.02  (0.45-2.33) | 0.97 | - | - |  |  |
| Family history^ | 1.30  (0.42-4.07) | 0.65 | - | - |  |  |
| Previous screening^^ | 1.31  (0.64-2.69) | 0.46 | - | - |  |  |
|  |  |  |  |  |  |  |
| **Psychological Factors** | | | | |  |  |
| Expected Anxiety | 3.94  (2.90-5.36) | <0.001 | 2.16  (1.44-3.33) | **<0.001** |  |  |
| Expected Burden | 4.36  (3.01-6.31) | <0.001 | 0.979  (0.784-2.42) | 0.087 |  |  |
| Expected Embarrassment | 3.44  (2.51-4.71) | <0.001 | 0.485  (0.485-1.53) | 0.064 |  |  |
| Expected Pain | 2.83  (1.95-4.12) | <0.001 | 0.725  (0.725-2.25) | 0.10 |  |  |
|  |  |  |  |  |  |  |
| **Procedural Factors** | | | | |  |  |
| US Procedure Length | 1.07  (0.96-1.20) | 0.23 | - | - |  |  |
| BMI | 1.07  (0.98-1.18) | 0.12 | - | - |  |  |
| Prostate Volume | 1.00  (0.97-1.03) | 0.12 | - | - |  |  |
| - Insufficient association on univariate analysis to continue in the multivariate analysis  * Most Deprived (Quintile 5) vs. Other, ** A-Level, Degree or Equivalent vs. lower/none  ¥ Employed vs. any other. ^ First or second degree relative with prostate cancer,  ^^ Defined as either PSA or DRE screening | | | | | | |
